# Conspiracy beliefs and vaccination intent for COVID-19 in an infodemic

**DOI:** 10.1371/journal.pone.0261559

**Published:** 2022-01-12

**Authors:** Ali Ghaddar, Sanaa Khandaqji, Zeinab Awad, Rawad Kansoun

**Affiliations:** 1 Observatory of Public Policies and Health, Beirut, Lebanon; 2 Department of Biomedical Sciences, Lebanese International University, Saida, Lebanon; 3 Department of Health Management & Policy, American University of Beirut, Beirut, Lebanon; 4 Department of Communication Arts, Lebanese International University, Beirut, Lebanon; Qazvin University of Medical Sciences, ISLAMIC REPUBLIC OF IRAN

## Abstract

**Background:**

The massive, free and unrestricted exchange of information on the social media during the Covid-19 pandemic has set fertile grounds for fear, uncertainty and the rise of fake news related to the virus. This “viral” spread of fake news created an “infodemic” that threatened the compliance with public health guidelines and recommendations.

**Objective:**

This study aims to describe the trust in social media platforms and the exposure to fake news about COVID-19 in Lebanon and to explore their association with vaccination intent.

**Methods:**

In this cross-sectional study conducted in Lebanon during July–August, 2020, a random sample of 1052 participants selected from a mobile-phone database responded to an anonymous structured questionnaire after obtaining informed consent (response rate = 40%). The questionnaire was conducted by telephone and measured socio-demographics, sources and trust in sources of information and exposure to fake news, social media activity, perceived threat and vaccination intent.

**Results:**

Results indicated that the majority of participants (82%) believed that COVID-19 is a threat and 52% had intention to vaccinate. Exposure to fake/ unverified news was high (19.7% were often and 63.8% were sometimes exposed, mainly to fake news shared through Watsapp and Facebook). Trust in certain information sources (WHO, MoPH and TV) increased while trust in others (Watsapp, Facebook) reduced vaccination intent against Covid-19. Believing in the man-made theory and the business control theory significantly reduced the likelihood of vaccination intent (Beta = 0.43; p = 0.01 and Beta = -0.29; p = 0.05) respectively.

**Conclusion:**

In the context of the infodemic, understanding the role of exposure to fake news and of conspiracy believes in shaping healthy behavior is important for increasing vaccination intent and planning adequate response to tackle the Covid-19 pandemic.

## Introduction

The rapid massive and unrestricted sharing of information on social media platforms during the COVID-19 pandemic has set fertile grounds for fear, uncertainty and the rise of fake news and conspiracy beliefs related to the virus. In this “infodemic” context, the role of exposure to social media and its associated conspiracy beliefs on vaccination intent is not well understood. Previous research about the influenza vaccine revealed that rumors and misconceptions shared throughout social media platforms spread panic, provoke anti-vaccine attitudes and beliefs, and raises mistrust in vaccines; ultimately leading to vaccine hesitancy and reduced vaccination intent [1]. Vaccine hesitancy has been associated with under-vaccination, particularly in children [2]; which is also accompanied by a rise in previously controlled diseases; for example, the CDC attributes the spread of measles to the reduction in measles vaccinations [3]. Furthermore, 41–50% of parents in the UK are unwilling to vaccinate their children [4], and half of the US population did not take the seasonal influenza vaccine [5].

While vaccine hesitancy has been declared a threat to global public health, the concept of public health vaccination programs should extend beyond the mere delivery of the vaccine to tackle the socio-environmental factors that increase COVD-19 vaccine confidence [6]. While the COVID-19 vaccine is being developed, its availability remains insufficient to ensure herd immunity without being trusted and accepted to the general population. It is thus important to explore the role of social media in shaping health behaviors related to acceptance of the COVID-19 vaccine to plan successful vaccination campaigns. Recent studies revealed that mistrust in vaccines and anti-vaccine beliefs reduced COVID-19 vaccination intent in the USA [7] and identified certain factors that explained vaccination intent, including the trust in healthcare systems [8] and risk perception [9].

Other studies used psychological to understand the psychological correlates of vaccination intent. The theory of planned behavior explained how vaccination intent is associated with fear, perceived infectability, attitude, knowledge, risk perception and previous influenza vaccination [10–12]. Furthermore, the protection motivation theory explained how perceived severity shaped the motivation for vaccination against Covid-19 [13, 14]. Despite the development and validation of scales for the measurement of willingness and reasons to get COVID-19 vaccination among different subgroups [15], the determinants of the intention to vaccinate are not yet well understood in the context of infodemics and belief in conspiracy theories.

Shortly after the WHO declaration of COVID-19 as a pandemic and a public health emergency [16], the unrestricted exchange of information on the web resulted in the saturation of social media with various verified and unverified information about the virus. An analysis of Tweets regarding the COVID-19 pandemic showed that 24.8% and 17.4% included misinformation or unverifiable information respectively [17]. In this crowd of news communicated on the web, it becomes hard to control the scientific merit of information, and it has been observed that information involving sensationalization and fear are more attractive, persuasive and more shared by users than dull accurate scientific news. While the increasing use of social media around the world presents an opportunity for its use as an essential and effective health communication strategy to provide the public with the needed information about the risky and preventive behaviors in such emerging pandemics, it also represents possible threat upon the dramatization and exaggeration of information, thus contributing to the so-called COVID-19 “infodemic” [18]. In fact, this infodemic involved problematic social media use and spread of fake news that were associated with several harms including anxiety, suicidal thoughts, distress and insomnia [19, 20].

First described in 2003 during the SARS outbreak, an infodemic (short for “information pandemic”), is “the overabundance of information that makes it difficult for people to find trustworthy sources for reliable guidance once needed” [21]. During the COVID-19 pandemic, the pace of internet searching for COVID-19 updates recorded a jump to 50–70%; in fact, 360 million related videos were uploaded on YouTube during a period of 30 days, 550 million COVID-19 related tweets were recorded in March of 2020, and articles published in Google Scholar has surpassed 19,000 since the pandemic commencement [21]. In this sense, credible news, fake news, and rumors spread amongst the public and “went viral”; similarly, as the disease spread. In this context, the WHO director-general Tedros Adhanom Ghebreyesus declared: “we’re not just fighting a pandemic; we’re fighting an infodemic” [22]. Infodemics have been reported during previous outbreaks, including typhus fever in Russia among Russian Jewish immigrants, as well as Hantavirus in Native Americans [23]. During the Ebola epidemic in the Democratic Republic of Congo, public health officials and international agencies realized the danger of the spread and manipulation of news that was disseminated through radio, WhatsApp; and as such, united their efforts to combat the spread of misinformation [24].

Besides its effect on COVID-19 vaccination intent, the spread of fake news prevents effective care and might threaten lives [25, 26]. Infodemics adversely affect the response to the pandemic and amplify its negative implications through a myriad of processes [27]. For instance, during the 2019 Ebola outbreak in the Democratic Republic of Congo, “misinformation was linked to violence, mistrust, social disturbances, and targeted attacks on healthcare providers” [28]. In addition, infodemics pose challenges for healthcare workers to find reliable information sources, and hinder rapid decision-making [29]. In fact, during the COVID-19 pandemic, several frightful non-evidence-based rumors have been widely disseminated across social media platforms, including: death forecasts; forecasts of shortages of ventilation ICU equipment; rumors about famous people contracting the disease; and warning against the use of anti-inflammatory drugs [30]. Notwithstanding, some of these rumors have even been adopted by scientific journals without sound evidence [30]. Furthermore, a recent study revealed that social media spreads misinformation regarding COVID-19; including: false claims (association between specific ethnic groups and the spread of COVID-19); conspiracy theories (e.g. 5G technology or mosquito bites transmitting COVID-19); and pseudoscientific health therapies (e.g. alleged cures, such as use of hydroxychloroquine and anti-malarials; drinking cow urine or disinfecting alcohol, which was responsible for the death of more than 5000 Iranians due to alcohol poisoning) [31, 32]. Van Prooijen & Douglas described the emotional and social determinants of conspiracy theories and identified feelings of anxiety and instability, fear and threat [33]. In unprecedented times of fear and uncertainty, such conspiracy theories may subsequently provide relieving justifications in times of crisis and vagueness without necessarily verifying information credibility.

On the other hand, the sense of fear attributed to the spread of rumors during the COVID-19 infodemic had negative repercussions on prevention and therapy. For instance, the massive media bombardment regarding alternative COVID-19 therapies has led people to storm pharmacies and buy stocks of available drugs such as hydroxychloroquine [34]. Also, fake news exchanged on social media platforms resulted in patients’ refusal to take medications such as ibuprofen to treat non-COVID-19 illnesses, and even refusal of being admitted into hospitals leading to lack of adequate treatment; and to patients with strokes delaying their hospital admission in fear of becoming infected with COVID-19 in a hospital setting [35].

The aim of this study is to explore the exposure to fake news about COVID-19 in Lebanon and to understand the public perceptions about conspiracy theories related to this virus. It also aims to explore the effect of conspiracy beliefs, perceived vulnerability and exposure to fake news on the intent to vaccinate against COVID-19.

## Methods

### Study design, procedure and data collection

This cross-sectional study was conducted among a simple random sample of persons living in Lebanon during the period of July–August, 2020, almost four months after the start of the COVID-19 epidemic in Lebanon. A structured questionnaire was developed by the research team based on literature reviews about exposure to social media and beliefs in conspiracy theories during pandemics. A phone survey was adopted in order to enforce prevention measures and maintain social distancing to protect data collectors from the transmission of COVID-19. An estimated sample size of 2653 participants were calculated based on a 99% confidence level with population = 6.8 million and an alpha-error of ±2.5%. The sampling was based on all probable mobile phone numbers in Lebanon (≥18 years old) that were taken from the Lebanese Ministry of Telecommunication website. The sample was randomly selected using the webpage: https://www.randomizer.org. The list of selected numbers was divided among four data collectors specially trained for the purpose of this research study. Data collectors called their assigned numbers in the list at least three times, and in case of no answer, replaced by the next number in the list. Data collectors read obtained verbal informed consent among those who accepted to participate. 1052 participants responded to the questionnaire, translating to a response rate of 40%.

### Ethical issues

The research followed the ethical principles of the Declaration of Helsinki. The questionnaire included a consent form that explains the research objectives and assures anonymity and confidentiality, and mentions that participation is completely voluntarily. The research was approved by the Institutional Review Board of the Lebanese International University (#LIUIRB- 200329-AG2).

### Variables

The questionnaire comprised a total of 20 close-ended questions and required 9 minutes on average to fill. It comprised of 5 sections: **Section 1:** 6 questions about socio- demographic information (sex, age, education, marital status and employment). **Section 2:** 5 questions about information sources and trust in these sources of information, exposure and type of fake news about COVID-19. Examples of questions in this section include: “What is/are your main source(s) of information about COVID-19? Tick all that apply: (World Health Organization (WHO); Ministry of Public Health (MoPH); WhatsApp; Facebook; Twitter; Instagram; TV/radio),” and “To what extent do you trust COVID-19 news from the below mentioned sources”? (answer options: never, rarely, sometimes and often) **Section 3:** 3 questions about social media activity related to COVID-19, including questions as: “Do you share, post or like news about COVID-19 on social media?, with answer options: never, rarely, often, and always. **Section 4:** 5 questions about perceived threat and conspiracy beliefs. Perceived threat was measured using the question: “I believe that Covid-19 is a threat to me and my family” (answer options: strongly agree neutral, disagree, and strongly disagree). Three pervasive conspiracy beliefs were explored asking participants: “Do you believe that COVID-19 is artificially man-made in a laboratory” (man- made theory), “Do you believe that COVID-19 is business tool to sell vaccines and medicaments” (business theory), and “Do you believe that COVID-19 is a tool for population control to reduce the number of elderly people”? (Population control theory). The answer options for questions in this section were: strongly agree neutral, disagree, and strongly disagree. **Section 5:** 1 question about the intent to vaccinate stating: “If there is a vaccine against corona-virus, do you intend on getting vaccinated?” with answer options: yes, no, unsure. The English and the Arabic versions of the questionnaire are available online as S1 and S2 Files.

### Measures

A pilot study was conducted on 30 individuals to check the reliability of the questionnaire. Modifications were applied to adjust the questionnaire to the context. The questionnaire reliability was assessed by calculating the alpha-Cronbach’s coefficients, which were acceptable for the main measures of the questionnaire. The values of alpha-Cronbach for each variable were respectively: trust in news from WHO = 0.67, trust in news from MoPH = 0.62, trust in news from Watsapp = 0.69, trust in news from Facebook = 0.66, exposure to fake news = 0.67, belief COVID-19 is exaggerated in the media = 0.71, belief COVID-19 is artificially man-made in the lab = 0.61, belief COVID-19 is a business tool to sell vaccines = 0.70, belief COVID-19 is a form of population control = 0.64.

### Statistical analysis

In the univariate analysis, descriptive statistics displayed the frequency and percentage of participants according to their level of trust in sources of information about COVID-19 (often, sometimes, rarely or never) and according to their conspiracy beliefs about COVID-19 (strongly agree, neutral, disagree and strongly disagree). Bivariate analysis compared vaccination intent (dependent variable with answers expressed as yes, no or unsure) according to socio-demographics and exposure and use of social media and beliefs in conspiracy theories about COVID-19 (independent variables), using the Chi-square test to explore statistically significant differences in the frequency of participants among the groups with alpha-error = 0.05. In the multivariate analysis, a stepwise logistic regression model was constructed with vaccination intent as dependent variable (Yes = 1; No or unsure = 0). The main assumptions for the logistic regression model were verified: absence of extreme outliers, absence of high multi-collinearity among the explanatory variables and linearity (Box-Tidwell test). Only variables with significant results in the bivariate analysis were entered in the multivariate logistic regression model as independent variables (sex, trust in news from different sources of information (MoPH, Watsapp, Facebook), exposure to fake news, belief that Covid-19 is exaggerated in the media, beliefs in conspiracy theories (Covid-is man-made, business theory and population control theory). Beta coefficients and confidence intervals were displayed to analyze the magnitude and the direction of associations and p-values of less than 0.05 were considered statistically significant.

## Results

### Socio-demographics

Socio-demographic information of participants is displayed in Table 1. The majority were female (66.3%), and were less than 40 years old (89.3%). Around half of participants were single (55.3%), undergraduates (50.6%) and unemployed (57%). Ninety-one percent of participants were Lebanese (Table 1).

**Table 1:** Socio-demographic information of participants.

Age	% (*n = 1052*)
18–24	41.7 (439)
25–40	47.6 (501)
41–60	8.6 (90)
>60	2.1 (22)
**Gender**	**% (*n = 1052)***
Male	31.8 (335)
Female	66.3 (697)
Prefer not to say	1.9 (20)
**Marital Status**	**% (*n = 1052)***
Single	55.3 (582)
Married	40.0 (421)
Divorced	3.2 (34)
Widowed	1.4 (15)
**Highest Degree Earned**	**% (*n = 1052)***
Primary	1.2 (13)
High School	24.1 (254)
Undergraduate	50.6 (532)
Graduate	20.6 (217)
Postgraduate	3.4 (36)
**Employment Status**	**% (*n = 1052)***
Employed	38.9 (409)
Unemployed	57.6 (606)
Retired	1.5 (16)
Disabled/Cannot Work	2.0 (21)
**Nationality**	**% (*n = 1048*)**
Lebanese	91.1 (955)
Non-Lebanese	8.9 (93)

### Sources of COVID-19 related information and trust

The majority of participants’ main source of information was from the Lebanese MOPH (58.4%), TV (51.5%), and the WHO (48.3%). No more than 40% of participants’ main source of information was from social media platforms (39.7% for Instagram and 37.9% for Facebook). The least used sources of COVID-19 information were the radio (6.2%), followed by Twitter (6.8%). Participants overall trusted the WHO the most when compared to other sources of information (54.8% as ‘often’), followed by the MOPH (43.3% as often and 37.3% as sometimes). 72% of participants also rated extent of trust in TV/radio news as either ‘often’ or ‘sometimes’. Overall, extent of trust in social media platforms was low; whereby WhatsApp had the lowest extent of trust (72.9% rated as ‘rarely’ or ‘never’), followed by 70.3% for Twitter, and 63.6% for Facebook.

### Exposure to wrong information, or fake/unverified news

Approximately 85% of participants reported been exposed to wrong information, or fake/unverified news (19.7% ‘often’ and 63.8% ‘sometimes’). Only 5.7% of participants reported never and 10.8% reported rarely have been exposed to wrong information, or fake/unverified news. When asked about the source of fake/unverified COVID-19 news, the lowest rated source of fake/unverified COVID-19 news was the WHO (12.4%) followed by the Lebanese MOPH (15.0%). The highest rated source of fake/unverified news was Whatsapp (73.7%), followed by Facebook/Instagram (60.6%). Describing exposure to fake news according to socio-demographics revealed that only age had significant correlation (p≤0.001), with around 85% in the younger age groups (18–24 and 25–40) reporting exposed in comparison to only 78% and 54% in the older age groups and with insignificant correlations between exposure with sex, employment, education and marital status. The most common types of fake/unverified news was regarding COVID-19 transmission modes (55.4%), exaggerating the harms/damage caused by the virus (48.5%), and theories claiming that COVID-19 is man-made (48.3%), harms of wearing masks and using disinfectants (20%), spread of the virus through 5G technology (26%), the effectiveness of medications and home remedies (33.7%).

### Sharing, posting, or liking COVID-19 news on social media

In general, participants were not active using social media when it comes to sharing, posting or liking posted information. Only around 1% of participants shared or posted news on social media (often and sometimes) and only around 4.5% “liked” (often or sometimes) posted news about COVID-19 on social media. The majority of participants never shared (69.6%), never posted (72.7%) and never liked (54.3%) these news. Among those who had activity on social media in sharing, posting or liking news about COVID-19, 68% never and 15.4% rarely shared information without checking originality or verifying news with expert sources. The majority of shared, posted, or liked information was related to personal preventive measures (53.6%, governmental updates (31.8%), scientific updates (e.g. virulence, infectivity, physical complications) (27.9%). A small percentage of the shared, posted or liked news was related to theories about the political origin of the virus (12.7%) and about theories about the political origin of the virus.

### Conspiracy beliefs about COVID-19

The majority (81.8%) of participants strongly agreed that COVID-19 is a threat to themselves and family, and only 0.4% strongly disagreed. Still, around a third of participants strongly agreed with and around half of participants was unsure/ neutral about the conspiracy theories about COVID-19: artificially made in a laboratory (man-made theory), a business tool to sell vaccines and medicaments (pharmaceutical industry theory) and population control theory.

### Vaccination intent

Just over half of participants (52.0%) have the intent to get vaccinated against COVID-19 upon availability of a vaccine and 33.3% were unsure about intent to get vaccinated. The rest (14.7%) were unwilling to get vaccinated.

### Bivariate analysis

Results of the Chi-square test showed that the frequency of vaccination intent was very close and did not differ significantly in the groups of marital status, age, education and occupation. Although single participants expressed slightly higher more intent to vaccination than ever married (54.1% vs. 49.4%) the difference was not statistically significant. Vaccination intent increased with trust in news from WHO, MoPH and radio/TV with statistically significant differences. Almost half of participants who often (59.3%) and sometimes (45.5%) trust WHO news expressed intent to vaccinate compared to only 9% and 17% did not intend to vaccinate respectively. The percentage of participants remained slightly higher in those who intend than do not intent to vaccinate between people who rarely (38.2% vs. 25%) and equal between those who never (37.8%) trust WHO news.

A similar picture was observed in the group differences (vaccination intent vs. non-intent) according to trust in news from WHO, radio/TV (Table 2). On the contrary, while vaccination intent did not differ significantly according to perception about the exaggeration of COVID-19 in the media it decreased with participant’s perception about being exposed to wrong/ wake or unverified news about COVID-19, and with two conspiracy theories (artificially man-made theory and business tool theory). Much higher percentages of participants with significant differences between the groups who believed that they were never (53.3%) or rarely (56.1%) exposed to fake news intended, compared to only around 14% who did not intend to vaccinate. Participants who disagreed with conspiracy theories had high reported intent to vaccinate 65% for man-made theory and 74.3% for business theory with lower percentages who intend to vaccinate as they agreed with conspiracy theories. Believing in population control theory did not have significant difference in percentages who intend or not to vaccinate.

**Table 2:** Vaccination intent vs. socio-demographic and social media exposure and use-related variables.

	COVID-19 vaccination intention			
	No	Yes	Unsure	p-value
***Gender***				0.05
Male	40 (11.9%)	188 (56.1%)	107 (31.9%)	
Female	109 (15.6%)	350 (50.2%)	238 (34.1%)	
***Marital status***				0.22
Single	78 (13.4%)	315 (54.1%)	189 (32.5%)	
Ever married	77 (16.4%)	232 (49.4%)	161 (34.3)	
***Age***				0.20
18–24	53 (12.1%)	227 (51.7%)	159 (36.2%)	
25–40	79 (15.8%)	241 (48.1%)	181 (36.1%)	
41–60	18 (20%)	39 (43.3%)	33 (36.7%)	
>60	5 (14.7%)	10 (52%)	7 (31.8%)	
***Educational***				0.38
Below Bachelor	111 (13.9%)	418 (52.3%)	270 (33.8%)	
Bachelor or Post-graduate	44 (17.4%)	129 (51%)	80 (31.5%)	
***Occupation***				0.73
Employed	56 (13.7%)	214 (52.3%)	139 (34%)	
Unemployed/ retired	99 (15.4%)	333 (51.8%)	211 (32.8%)	
***Belief that Covid-19 is a threat***				0.53
Strongly agree	126 (17.9%)	452 (64.2%)	126 (17.9%)	
Unsure	24 (18.2%)	84 (63.6%)	24 (18.2%)	
Disagree	3 (18.8%)	10 (62.4%)	3 (18.8%)	
Strongly disagree	2 (40%)	1 (20%)	2 (40%)	
***Trust in news from WHO***				≤0.001
Never	28 (37.8%)	28 (37.8%)	18 (24.3%)	
Rarely	19 (25%)	29 (38.2%)	28 (36.8%)	
Sometimes	56 (17.2%)	148 (45.5%)	121 (37.2%)	
Often	52 (9%)	342 (59.3%)	183 (31.7%)	
***Trust in news from MoPH***				≤0.001
Never	31 (33.3%)	35 (37.6%)	27 (29%)	
Rarely	25 (22.3%)	41 (36.6%)	46 (41.1%)	
Sometimes	59 (15.1%)	197 (50.3%)	136 (34.7%)	
Often	40 (8.8%)	274 (60.2%)	141 (31%)	

As for conspiracy beliefs, the Chi-square test showed that 53.7% of males vs. 44.5 of females strongly agreed that COVID-19 is exaggerated in the media, and 38.8% of males vs. 32.3 of females strongly believed that COVID-19 is a tool for population control, showing significant association of these believes with sex (p = 0.03 & p = 0.04, respectively). Furthermore, 51.2% of unemployed vs. 42.3% of employed strongly agreed with the fact that COVID-19 is exaggerated in the media (p = 0.01). Both sharing news and posting news about COVID-19 on social media were negatively associated with belief in that Covid-19 is a threat (significant associations) (p = 0.02 & p = 0.003, respectively). Those who strongly believe in the threat of COVID-19 less often share and post news in social media. For instance, while 80.9% and 82.9% of respondents who strongly believe in the threat of COVID-19 never share and post news on social media, compared to a lower percentage of respondents who sometimes share and post news (63.2% and 50%).

### Logistic regression

Results of the logistic regression model showed that being female, trust in Facebook, exposure to fake news, belief in man-made theory and business control theory significantly reduced the likelihood of intent to vaccinate and that trust in news from WHO, MoPH, and WhatsApp increased the likelihood to vaccinate (Table 3). However, although exposure to fake news reduced vaccination intent and belief in the population control theory increased vaccination intent, the reported associations were statistically insignificant (p = 0.39 & p = 0.53, respectively). The strongest magnitudes of association were noted for trust in news from MoPH (trusting this news increased vaccination intent 0.53 points; p = 0.003) and belief in the business tool theory (strongly agreeing with this theory reduced the likelihood for vaccination by 0.43 points; p = 0.01).

**Table 3:** Logistic regression with vaccination intent as dependent variable (yes = 1; no = 0) and gender, trust in social media and conspiracy theories as independent variables*.

Variable			
	Beta	p-value	C.I.
**Female**	-.313	0.016	(0.57;0.94)
**Trust in news from WHO** (Often or sometimes)	0.425	0.037	(1.026; 2.28)
**Trust in news from MoPH** (Often or sometimes)	0.53	0.003	(1.195;2.45)
**Trust in news from Whatsapp** (Often or sometimes)	0.40	0.023	(1.05;2.12)
**Trust in news from Facebook** (Often or sometimes)	-0.29	0.070	(0.54;1.02)
**Exposure to wrong, fake or unverified news about Covid-19** (Often or sometimes)	-.149	0.395	(0.61;1.21)
**Belief COVID-19 is exaggerated in the media** (Strongly agree)	-.356	0.007	(1.10;1.85)
**Belief COVID-19 is artificially man-made in the lab (Strongly agree)**	-0.29	0.05	(0.66;0.78)
**Belief COVID-19 is a business tool to sell vaccines (Strongly agree)**	-0.43	0.02	(0.75;0.91)
**Belief COVID-19 is a form of population control (Strongly agree)**	0.108	0.53	(0.65;1.23)

*only variables with significant results in the bi-variate analysis entered in the model.

## Discussion

Results indicated that although the majority of participants believed that COVID-19 is a threat, only half of them reported intention to vaccinate, while only one third believed in one or more conspiracy theories related to COVID-19. Results also implied that female sex, trust in Facebook, exposure to fake news, belief in man-made theory and business control theory significantly reduced the likelihood of intent to vaccinate and that trust in news from WHO, MoPH, and WhatsApp increased the intention to vaccinate.

### Comparison with other studies

Results revealed that the majority of participants’ main source of information was from the MoPH (58.4%) and TV (51.5%), in line with previous studies that discussed how higher levels of institutional trust are expected in pandemics [36], and with another study in the same context in Lebanon that showed TV as the main trusted media source [37]. Our study results show that around a third of the study population depend on social media platforms including WhatsApp (30.2%), Facebook (37.9%) and Instagram (39.7%) as a main source of COVID-19 information. Sharing, posting, or liking COVID-19 news on social media though was not common among participants, even to a lower extent than similar studies in the same country [37].

The percentage of study participants who intend to vaccinate in our study (52% certain, 33% unsure and 15% no) was very similar to results observed in other contexts among the general population in Malaysia (86.1%) [38], in the USA [7] and in Italy [39]. Furthermore, results of this study confirmed that vaccination intent increased with the trust in news from health institutions (WHO, MOPH, and radio/TV), in consistence with other studies which have shown that acceptability of a COVID-19 vaccine among adults in the USA was influenced by trust in institutions [40], that lack of trust in vaccine related to anti-vaccine attitudes or beliefs in the USA [7], and that trust in the health system was significant determinants of a COVID-19 vaccine acceptance in the Middle East [41], and that trust in research and in vaccines predicted intention to vaccinate among general population in Italy [39]. In contrast to other studies that highlighted younger age and lower educational as a predictor to vaccine hesitancy [7], age and education had no significant association with vaccination intent in our study. Results concerning the absence of significant association between perceived threats of COVID-19 with intent to vaccinate, however, contradict those of previous studies that showed that perception of disease risk increases vaccination intent [40] in the general population, and that perceived risk of disease increased acquiescence to COVID-19 vaccination among healthcare workers [41].

Furthermore, this study revealed that vaccination intent significantly decreased with perception of exposure to wrong/fake or unverified news, in agreement with findings from other studies that described how vaccine hesitancy is shaped by the spread of fake news and misinformation on social media [20, 42]. On the other hand, around a third of participants strongly believed in one or more conspiracy theories related to COVID-19, and that the belief in two of the conspiracy theories (man-made theory and business control theory) were significantly negatively associated with vaccination intent. Conspiracy theories have been reported in previous pandemics, with the possibility of affecting compliance to preventive measures and intention to vaccinate [43–47].

### Limitations

Certain limitations should be taken into account upon interpreting the results of this study. Firstly, it is important to recognize that all measured exposure and outcome variables are self- reported which creates a possible source of information bias, especially when it is previously discussed that reported vaccination intent seems to be underestimated in surveys [48]. In addition, the current study is challenged by the character of the study population and its design. The cross-sectional design of the study poses some limitations to the validity of its results due to the difficulty to exclude confounders. Furthermore, almost all participants had at least a high-school education, the fact that could limit the generalizability of the results due to possible selection bias. Nevertheless, it could be argued that the majority of the Lebanese population is literate and thus the sample is more or less representative to the general population. Further bias could have also occurred due to the fact that the study did not take into consideration measuring the economic situation of the participants, although it is previously discussed how gross domestic product creates Covid-19 vaccination inequity [49]. This is the first study that explored vaccination intent in relation to belief in conspiracy theories in the context of infodemics.

### Policy implications

The fact that the majority (85%) of participants reported exposure to fake/unverified news raises concerns for fast diffusion of information through social media causing undesired health behavior, panic, fear and anxiety from COVID-19 [50–53]. In other words, trust in social media has a negative effect on knowledge and adherence to preventive practices such as social distancing [54].

In attempt to mitigate the undesired effect of infodemic on healthy behavior and preventive practices, the WHO has recently issued a framework for infodemic management in health emergencies to address the adverse implications of COVID-19 infodemic. The framework enforces five core policy areas that includes “assuring reliable detection system of the information flow, especially misinformation and disinformation dynamics within communities by health sector entities” [55]. In response, in April of 2020, WhatsApp restricted message forwarding to only a single contact if a message had been shared with 5 users [56] as a means to control the spread of misinformation [57]. Facebook put warning labels on approximately 50 million COVID-19-related articles to also help in combating COVID-19 mis/disinformation, in addition to removing harmful content, and a new notification screen that provides context to the articles that are shared [58, 59]. Instagram has also taken a step in the same direction through “removing COVID-19 accounts from account recommendations, removing COVID-19 related content from the Explore page unless posted by a credible health organization, and downranking content that has been rated false by third-party fact checkers” [60]. These active reforms of social media platforms in collaboration with the WHO should be emphasized to continuously monitor and respond to fake news [61]. Similarly, the Royal Society of Public Health in England warns about the harms of spreading fake news in social media and urged for certifying the social media platforms that relying on credible source of information and not on popularity [62]. One should take into consideration to question the reliability of information not only on social media but even that information disseminated through some scientific journals with the pressure to publish and use of unverified non-peer reviewed pre-prints [63].

### Future research and recommendations

The issue of unrestricted sharing of fake news through social media is a major public health concern, especially due to the harms it causes in adversely shaping preventive behavior and inducing vaccine hesitancy. The danger of the infodemic should be acknowledged and firm measures should be put forth; this includes considering strict measures such as prison sentences issuance, as that taken by the government in Peru against those who create and share fake news [64]. Furthermore, when it comes to designing prevention strategies, it would have been interesting to explore the engagement of users in social media platforms according to the nature of messages and to explore the exposure and the vulnerability of the population to fake news and their engagement in social media networks according to socio-demographics, values and belief system to compare with results observed using content analysis of posts on social media platforms [65]. However, engagement in our sample (posting and liking information) was surprisingly low and thus belief in conspiracy was expressed in terms of self-reported data on a Likert scale and not in terms of actual posting, liking or sharing information. Moreover, it would have been of value to study more in depth the drivers of believing in the conspiracy theories as suggested by previous research [66], however our study did not show significant correlation with socio-demographic groups except for age. While addressed in a recent study in Italy [67], the role of mechanism of search behavior in social media is still not clearly understood; and this could be a topic of importance to discuss in future studies. Moreover, future studies could try to further understand the role of the socio-political factors in shaping willingness to vaccinate, as previously reported in a study about how politicization affect COVID-19 vaccination intent in France [68].

## Supporting information

S1 File Survey (English version). (PDF)

S2 File Survey (Arabic version). (PDF)

## References

[1] Schmid P, Rauber D, Betsch C, Lidolt G, Denker ML. Barriers of Influenza Vaccination Intention and Behavior—A Systematic Review of Influenza Vaccine Hesitancy, 2005–2016. PLoS One. 2017; doi: 10.1371/journal.pone.0170550 28125629 PMC5268454

[2] Tomljenovic H, Bubic A, Erceg N. It just doesn’t feel right—the relevance of emotions and intuition for parental vaccine conspiracy beliefs and vaccination uptake. Psychology & health. 2020; 10.1080/08870446.2019.1673894 31588791

[3] Yang L, Grenfell BT, Mina MJ. Measles vaccine immune escape: Should we be concerned? Eur J Epidemiol. 2019; doi: 10.1007/s10654-019-00574-7 31676977

[4] Campbell H, Edwards A, Letley L, Bedford H, Ramsay M, Yarwood J. Changing attitudes to childhood immunisation in English parents. Vaccine. 2017. 10.1016/j.vaccine.2017.03.089 28442229

[5] CDC. Flu Vaccination Coverage, United States, 2018–19 Influenza Season. 2019; https://www.cdc.gov/flu/fluvaxview/coverage-1819estimates.htm

[6] Harrison EA, Wu JW. Vaccine confidence in the time of COVID-19. Eur J Epidemiol. 2020; 10.1007/s10654-020-00634-3 32318915 PMC7174145

[7] Fisher KA, Bloomstone SJ, Walder J, Crawford S, Fouayzi H, Mazor KM. Attitudes Toward a Potential SARS-CoV-2 Vaccine: A Survey of U.S. Adults. Annals of internal medicine. 2020; 10.7326/M20-3569 PMC7505019 32886525

[8] Ahorsu D. K., Lin C.-Y., Yahaghai R., Alimoradi Z., Broström A., Griffiths M. D., et al. (2021). The mediational role of trust in the healthcare system in the association between generalized trust and willingness to get COVID-19 vaccination in Iran. Human Vaccines & Immunotherapeutics. doi: 10.1080/21645515.2021.1993689 34715009 PMC8920226

[9] Kukreti S., Lu M.-Y., Lin Y.-H., Strong C., Lin C.-Y., Ko N.-Y., et al. (2021). Willingness of Taiwan’s healthcare workers and outpatients to vaccinate against COVID-19 during a period without community outbreaks. Vaccines, 9(3), 246. doi: 10.3390/vaccines9030246 33808950 PMC8000386

[10] Yahaghi R, Ahmadizade S, Fotuhi R, Taherkhani E, Ranjbaran M, Buchali Z, et al. Fear of COVID-19 and Perceived COVID-19 Infectability Supplement Theory of Planned Behavior to Explain Iranians’ Intention to Get COVID-19 Vaccinated. Vaccines. 2021; 9(7):684. 10.3390/vaccines9070684 34206226 PMC8310138

[11] Fan Chia-Wei, Chen I-Hua, Ko Nai-Ying, Yen Cheng-Fang, Lin Chung-Ying, Griffiths Mark D. et al. (2021) Extended theory of planned behavior in explaining the intention to COVID-19 vaccination uptake among mainland Chinese university students: an online survey study, Human Vaccines & Immunotherapeutics, 17:10, 3413–3420, doi: 10.1080/21645515.2021.1933687 34170792 PMC8437493

[12] Ullah I., Lin C.-Y., Malik N. I., Wu T.-Y., Araban M., Griffiths M. D., et al. (2021). Factors affecting Pakistani young adults’ intentions to uptake COVID-19 vaccination: An extension of the theory of planned behavior. Brain and Behavior. 10.1002/brb3.2370 34543522 PMC8613438

[13] Wang PW, Ahorsu DK, Lin CY, et al. Motivation to Have COVID-19 Vaccination Explained Using an Extended Protection Motivation Theory among University Students in China: The Role of Information Sources. Vaccines (Basel). 2021;9(4):380. Published 2021 Apr 13. doi: 10.3390/vaccines9040380 33924604 PMC8070343

[14] Huang P.-C., Hung C.-H., Kuo Y.-J., Chen Y.-P., Ahorsu D. K., Yen C.-F., et al. (2021). Expanding Protection Motivation Theory to explain willingness of COVID-19 vaccination uptake among Taiwanese university students. Vaccines, 9, 1046. doi: 10.3390/vaccines9091046 34579283 PMC8473221

[15] Yeh Y.-C., Chen I.-H., Ahorsu D. K., Ko N.-Y., Chen K.-L., Li P.-C., et al. (2021). Measurement invariance of the Drivers of COVID-19 Vaccination Acceptance Scale: Comparison between Taiwanese and mainland Chinese-speaking populations. Vaccines, 9(3), 297. doi: 10.3390/vaccines9030297 33810036 PMC8004810

[16] Schaffer-DeRoo S, Pudalov NJ, Fu LY. Planning for a COVID-19 Vaccination Program. JAMA.2020; 10.1001/jama.2020.8711 32421155

[17] Kouzy R, Abi Jaoude J, Kraitem A, El Alam MB, Karam B, Adib E, et al. Coronavirus Goes Viral: Quantifying the COVID-19 Misinformation Epidemic on Twitter. Cureus; 10.7759/cureus.7255 32292669 PMC7152572

[18] Wang Y, McKee M, Torbica A, Stuckler D. Systematic Literature Review on the Spread of Health-related Misinformation on Social Media. Social science & medicine (1982). 2019; 10.1016/j.socscimed.2019.112552 31561111 PMC7117034

[19] Pramukti I., Strong C., Sitthimongkol Y., Setiawan A., Pandin M. G. R., Yen C.-F., et al. (2020). Anxiety and suicidal thoughts during the COVID-19 pandemic: A cross-country comparison among Indonesian, Taiwanese, and Thai university students. Journal of Medical Internet Research, 22(12), e24487. doi: 10.2196/24487 33296867 PMC7772053

[20] Lin C.-Y., Broström A., Griffiths M. D., & Pakpour A. H. (2020). Investigating mediated effects of fear of COVID-19 and COVID-19 misunderstanding in the association between problematic social media use and distress/insomnia. Internet Interventions, 21, 100345. doi: 10.1016/j.invent.2020.100345 32868992 PMC7449889

[21] PAHO. Understanding the Infodemic and Misinformation in the Fight Against COVID-19. 2020. https://iris.paho.org/bitstream/handle/10665.2/52052/Factsheet-infodemic_eng.pdf?sequence=14&isAllowed=y

[22] WHO. Munich Security Conference. 2020b. https://www.who.int/director-general/speeches/detail/munich-security-conference

[23] Patel MP, Kute VB, Agarwal SK, COVID-19 Working Group of Indian Society of Nephrology. "Infodemic" COVID 19: More Pandemic than the Virus. Indian journal of nephrology. 2020; 10.4103/ijn.IJN_216_20 PMC7470201 33013069

[24] Spinney L. ‘Fighting Ebola is hard. In Congo, fake news makes it harder’. Sciencemag. 2020. https://www.sciencemag.org/news/2019/01/fighting-ebola-hard-congo-fake-news-makes-it-harder

[25] Strekalova YA. Health Risk Information Engagement and Amplification on Social Media: News About an Emerging Pandemic on Facebook. Health Education & Behavior. 2017; 10.1177/1090198116660310 27413028

[26] Witte K, Allen M. A Meta-Analysis of Fear Appeals: Implications for Effective Public Health Campaigns. Health Education & Behavior. 2000; 10.1177/109019810002700506 11009129

[27] UN. UN tackles ‘infodemic’ of misinformation and cybercrime in COVID-19 crisis. 2020. https://www.un.org/en/un-coronavirus-communications-team/un-tackling-‘infodemic’-misinformation-and-cybercrime-covid-19

[28] WHO. Ebola virus disease–Democratic Republic of the Congo. 2019. https://www.who.int/csr/don/28-november-2019-ebola-drc/en/

[29] WHO. Managing the COVID-19 Infodemic: Promoting healthy behaviors and mitigating the harm from misinformation and disinformation. 2020c. https://www.who.int/news-room/detail/23-09-2020-managing-the-covid-19-infodemic-promoting-healthy-behaviours-and-mitigating-the-harm-from-misinformation-and-disinformation

[30] Orso D, Federici N, Copetti R, Vetrugno L, Bove T. Infodemic and the spread of fake news in the COVID-19-era. Eur J Emerg Med. 2020; 10.1097/MEJ.0000000000000713 32332201 PMC7202120

[31] Naeem SB, Bhatti R, Khan A. An exploration of how fake news is taking over social media and putting public health at risk. Health Information & Libraries Journal. 2020; 10.1111/hir.12320 32657000 PMC7404621

[32] Trew B. Coronavirus: Hundreds dead in Iran from drinking methanol amid fake reports it cures disease. Independent. 2020; https://www.independent.co.uk/news/world/middle-east/iran-coronavirus-methanol-drink-cure-deaths-fake-a9429956.html

[33] van Prooijen JW, Douglas KM. Belief in conspiracy theories: Basic principles of an emerging research domain. European journal of social psychology. 2018; 10.1002/ejsp.2530 30555188 PMC6282974

[34] Pantaleón D. Tratamiento Saca a Cinco de Cuidados Intensivos. Listín Diario. 2020. https://listindiario.com/la-republica/2020/03/28/610777/tratamiento-saca-a-cinco-de-cuidados-intensivos

[35] Diegoli H, Magalhães PSC, Martins SCO, et al. Decrease in Hospital Admissions for Transient Ischemic Attack, Mild, and Moderate Stroke During the COVID-19 Era. Stroke. 2020. 10.1161/STROKEAHA.120.030481 PMC7302100 32530738

[36] Esaiasson P, Sohlberg J, Ghersetti M, Johansson B. How the coronavirus crisis affects citizen trust in institutions and in unknown others: Evidence from ‘the Swedish experiment’. European Journal of Political Research. 2020; 10.1111/1475-6765.12419

[37] Melki J, Hitti E, Abou Zeid M, El Takach A, Hadid A, Ghandour L, et al. Mitigating Infodemics: A working paper on media and communication uses in Lebanon during the COVID-19 pandemic. Lebanese American University, Beirut, Lebanon. 2020. http://imrt.lau.edu.lb/sites/default/files/Latest%20Research/Mitigating%20Infodemics%20IMRT%20Web.pdf

[38] Wong LP, Alias H, Wong PF, Lee HY, AbuBakar S. The use of the health belief model to assess predictors of intent to receive the COVID-19 vaccine and willingness to pay. Human vaccines & immunotherapeutics. 2020; 10.1080/21645515.2020.1790279 32730103 PMC7553708

[39] Palamenghi L, Barello S, Boccia S, Graffigna G. Mistrust in biomedical research and vaccine hesitancy: the forefront challenge in the battle against COVID-19 in Italy. 2020; 10.1007/s10654-020-00675-8 PMC7431109 32808095

[40] Reiter PL, Pennell ML, Katz ML. Acceptability of a COVID-19 vaccine among adults in the United States: How many people would get vaccinated?. Vaccine. 2020; 10.1016/j.vaccine.2020.08.043 32863069 PMC7440153

[41] Dror AA, Eisenbach N, Taiber S, Morozov NG, Mizrachi M, Zigron A, et al. Vaccine hesitancy: the next challenge in the fight against COVID-19. Eur J Epidemiol. 2020; 10.1007/s10654-020-00671-y 32785815 PMC8851308

[42] Aquino F, Donzelli G, De Franco E, Privitera G, Lopalco PL, Carducci A. The web and public confidence in MMR vaccination in Italy. Vaccine. 2017; 10.1016/j.vaccine.2017.07.029 28736200

[43] Imhoff R, Lamberty P. A Bioweapon or a Hoax? The Link Between Distinct Conspiracy Beliefs About the Coronavirus Disease (COVID-19) Outbreak and Pandemic Behavior. 2020; 10.1177/1948550620934692 PMC7342934 38602949

[44] Romer D, Jamieson KH. Conspiracy theories as barriers to controlling the spread of COVID-19 in the U.S. Soc Sci Med. 2020; 10.1016/j.socscimed.2020.113356 32967786 PMC7502362

[45] Smallman S. Whom do you trust? Doubt and conspiracy theories in the 2009 influenza pandemic. Journal of International & Global Studies. 2015; https://pdxscholar.library.pdx.edu/is_fac/12/

[46] Cohen D, Carter P. Conflicts of interest. WHO and the pandemic flu "conspiracies". BMJ. 2010; 10.1136/bmj.c2912 20525679

[47] Setbon M, Raude J. Factors in vaccination intention against the pandemic influenza A/H1N1. European Journal of Public Health. 2010; 10.1093/eurpub/ckq054 20444821 PMC7314046

[48] Rieger MO. Willingness to vaccinate against COVID-19 might be systematically underestimated. Asian J Soc Health Behav 2021;4:81–3

[49] Alimoradi Z, Lin CY, Pakpour AH. Coronavirus disease-19 vaccine inequity and gross domestic product. Asian J Soc Health Behav 2021;4:129–30

[50] Ahmad AR, Murad HR. The Impact of Social Media on Panic during the COVID-19 Pandemic in Iraqi Kurdistan: Online Questionnaire Study. Journal of medical Internet research. 2020; 10.2196/19556 32369026 PMC7238863

[51] Greene C, Murphy G. Can fake news really change behaviour? Evidence from a study of COVID-19 misinformation. 2020; doi: 10.31234/osf.io/qfnm3 34110860

[52] Ireton C, Posetti J. Journalism, fake news & disinformation: handbook for journalism education and training. UNESCO. 2018; https://digitallibrary.un.org/record/1641987?ln=en

[53] Zhou X, Zafarani R. Fake news: A survey of research, detection methods, and opportunities. 2018; https://arxiv.org/abs/1812.00315

[54] Fridman I, Lucas N, Henke D, Zigler CK. Association Between Public Knowledge About COVID-19, Trust in Information Sources, and Adherence to Social Distancing: Cross-Sectional Survey. JMIR public health and surveillance. 2020; 10.2196/22060 PMC7511226 32930670

[55] Tangcharoensathien V, Calleja N, Nguyen T, Purnat T, D’Agostino M, Garcia-Saiso S., et al. Framework for managing the COVID-19 infodemic: methods and results of an online, crowdsourced WHO technical consultation. Journal of medical Internet research. 2020; 10.2196/19659 32558655 PMC7332158

[56] Newton C. WhatsApp puts new limits on the forwarding of viral messages. The Verge. 2020; https://www.theverge.com/2020/4/7/21211371/whatsapp-message-forwarding-limits-misinformation-coronavirus-india

[57] Singh M. Whatsapp introduces new limit on message forwards to fight spread of misinformation. 2020; https://techcrunch.com/2020/04/07/whatsapp-rolls-out-new-limit-on-message-forwards/

[58] Statt, N. Facebook will now show a warning before you share articles about COVID-19. The Verge. 2020; https://www.theverge.com/2020/8/12/21365305/facebook-covid-19-warning-notification-post-misinformation

[59] Rosen, G. An Update on Our Work to Keep People Informed and Limit Misinformation About COVID-19. Facebook. 2020; https://about.fb.com/news/2020/04/covid-19-misinfo-update/

[60] Instagram. COVID-19 Announcement. 2020; https://about.instagram.com/blog/announcements/coronavirus-keeping-people-safe-informed-and-supported-on-instagram

[61] Zarocostas J. (2020). How to fight an infodemic. Lancet. 10.1016/S0140-6736(20)30461-X 32113495 PMC7133615

[62] RSPH. Joint response from the Royal Society for Public Health and the APPG on Social Media to the government consultation on Online Harms White Paper. 2018; https://www.rsph.org.uk/static/uploaded/64bdfc80-7296-4ed7-94771778d1bd5d17.pdf

[63] Casigliani V, De Nard F, De Vita E, Arzilli G, Grosso FM, Quattrone F, et al. Too much information, too little evidence: is waste in research fuelling the covid-19 infodemic?. BMJ. 2020; 10.1136/bmj.m2672 32631897

[64] Alvarez-Risco A, Mejia CR, Delgado-Zegarra J, Del-Aguila-Arcentales S, Arce-Esquivel AA, Valladares-Garrido MJ, et al. The Peru Approach against the COVID-19 Infodemic: Insights and Strategies. The American journal of tropical medicine and hygiene. 2020; 10.4269/ajtmh.20-0536 32500853 PMC7410469

[65] Ali K, Zain-ul-abdin K, Li C, Johns L, Ali AA, Carcioppolo N. Viruses Going Viral: Impact of Fear-Arousing Sensationalist Social Media Messages on User Engagement. Science Communication. 2019; 10.1177/1075547019846124

[66] Ahmed W, Vidal-Alaball J, Downing J, López Seguí F. COVID-19 and the 5G Conspiracy Theory: Social Network Analysis of Twitter Data. Journal of medical Internet research. 2020; 10.2196/19458 32352383 PMC7205032

[67] Rovetta A, Bhagavathula AS, Castaldo L. Modeling the Epidemiological Trend and Behavior of COVID-19 in Italy. Cureus 2020; 10.7759/cureus.9884 PMC7505530 32968550

[68] COCONEL Group. A future vaccination campaign against COVID-19 at risk of vaccine hesitancy and politicisation. The Lancet. Infectious diseases. 2020; 10.1016/S1473-3099(20)30426-6 32445713 PMC7239623

