# Supplementary material for: Conspiracy beliefs and vaccination intent for COVID-19 in an infodemic
Source: PLoS One. 2022 Jan 12;17(1):e0261559. doi: 10.1371/journal.pone.0261559 (PMC8754330; doi:10.1371/journal.pone.0261559)
Supplement: S1 File — (PDF) [file pone.0261559.s001.pdf]

COVID-19 Infodemic Survey

Dear participant:

The Observatory of Public Policies and Health is conducting a study about the current COVID-19 related infodemic and the massive flow of fake information to the general public. The below questionnaire will be used to assess the public's exposure to fake news and its implications on behaviors and perceptions about COVID-19.
Please note that participation is voluntary and that your decision to participate or not will not affect you in any way. In addition, all the answers you provide will be handled confidentially.
Results from this study will be used to inform the national response to control COVID-19 infodemic.

There are no direct or indirect risks associated with completing this questionnaire, and your identity will be kept anonymous.
Please note that by responding to this survey, you are consenting to participate in this study.
If you have concerns about the study or questions about your rights as a participant, you can contact the Observatory of Public Policies and Health Office by phone on +961 71 729795

* Required

1. 1. Age: *

Mark only one oval.

- 18-24 years
- 25-40 years
- 41-60 years
- > 60 years

2. 2. Gender: *

Mark only one oval.

- Male
- Female
- Prefer not to say

3. 3. Marital Status: *

Mark only one oval.

- Single
- Married
- Divorced
- Widowed

4. 4. Highest degree earned: *

Mark only one oval.

- Primary
- High School
- Undergraduate
- Graduate
- Post Graduate

5. 5. Employment Status: *

Mark only one oval.

- Employed
- Unemployed
- Retired
- Disabled/cannot work

6. 6. Nationality: *

Mark only one oval.

- Lebanese
- Non-Lebanese

Information Sources

7. 7. What is/are your main source(s) of information about COVID-19? Tick all that apply. *

Check all that apply.

- [ ] WHO website
- [ ] Ministry of Public Health Website
- [ ] WhatsApp
- [ ] Facebook
- [ ] Twitter
- [ ] Instagram
- [ ] TV News
- [ ] Radio News

Other: [ ] ______________________

8. 8. To what extent do you trust COVID-19 news from the below mentioned sources? *

Check all that apply.

	Never	Rarely	Sometimes	Often
WHO website	☐	☐	☐	☐
Ministry of Public Health Website	☐	☐	☐	☐
WhatsApp	☐	☐	☐	☐
Facebook, Instagram	☐	☐	☐	☐
Twitter	☐	☐	☐	☐
TV/ Radio News	☐	☐	☐	☐

9. 9. Do you think you have been exposed to wrong information/ fake or unverified news about Covid19? *

Mark only one oval.

- Never
- Rarely
- Sometimes
- Often

10. 10. If you answered “rarely, sometimes or often” to the Question 9, what was/were the source(s) of the fake and\or unverified news? *

Check all that apply.

- [ ] WHO website
- [ ] Ministry of Public Health Website
- [ ] WhatsApp
- [ ] Facebook, Instagram
- [ ] Twitter
- [ ] TV/ Radio News
- [ ] N/A

11. 11. If you answered “rarely, sometimes or often” to the Question 9: what was the type of the fake and\or unverified news?

Check all that apply.

- [ ] Wrong information about the transmission modes
- [ ] Exaggerating the harms/ damage caused by Covid-19
- [ ] Theories about Covid-19 being man-made
- [ ] Describing the harms caused by wearing masks and using disinfectants
- [ ] Theories about the spread of the virus through 5G technology
- [ ] The effectiveness of certain drugs and medications
- [ ] The effectiveness of home remedies using nutrition
- [ ] N/A

Other: [ ] ____________________

Social media activity

12. 12. Do you share, post or like news about COVID-19 on social media? *

Check all that apply.

	Never	Rarely	Sometimes	Often
Share	☐	☐	☐	☐
Post	☐	☐	☐	☐
Like	☐	☐	☐	☐

13. 13. If you answered "rarely, sometimes or often" to any of the above, the information shared, posted or liked is related to: Tick all that apply *

Check all that apply.

- ☐ Personal Preventive Measures (such as wearing masks and disinfecting items...)
- ☐ Governmental Updates (e.g. lock-down, national COVID-19 cases)
- ☐ COVID-19 Scientific Updates (e.g. virulence, infectivity, physical complications)
- ☐ Theories related to the political origin of COVID-19
- ☐ I do not remember my post or share
- ☐ N/A

14. 14. Do you share information about COVID-19 without checking the originality or verifying it with expert sources? *

Mark only one oval.

- ◯ Never
- ◯ Rarely
- ◯ Often
- ◯ Always

Covid-19 conspiracy theories

15. 15. I believe that COVID-19 is (Tick all that apply): *

Check all that apply.

	Strongly Agree	Neutral/Unsure	Disagree	Strongly Disagree
Exaggerated in the media (virulence, mortality, etc.)	☐	☐	☐	☐
Artificially made in a laboratory	☐	☐	☐	☐
A business tool to sell vaccines and medicaments	☐	☐	☐	☐
A tool for population control and demographic changes to reduce the number of elderly people	☐	☐	☐	☐
A threat to me and my family	☐	☐	☐	☐

Vaccination intent

16. 16. If there is a vaccine against corona-virus, do you intend on getting vaccinated? *

Mark only one oval.

- ( ) Yes
- ( ) No
- ( ) Not sure

17. THANK YOU FOR YOUR PARTICIPATION

This content is neither created nor endorsed by Google.

Google Forms
