# Supplementary material for: Conspiracy beliefs and vaccination intent for COVID-19 in an infodemic
Source: PLoS One. 2022 Jan 12;17(1):e0261559. doi: 10.1371/journal.pone.0261559 (PMC8754330; doi:10.1371/journal.pone.0261559)
Supplement: S2 File — (PDF) [file pone.0261559.s002.pdf]

دراسة "وباء المعلومات" في زمن الكورونا

عزيزي(تي) المشارك(ة)

يجري مرصد السياسات العامة والصحة دراسة حول وباء المعلومات الحالي المتعلق بـمرض فيروس كورونا المستجد (COVID-19) والتدفق الهائل للمعلومات المزيفة إلى عامة الناس. سيتم استخدام الاستبيان أدناه لتقييم تعرض الجمهور للأخبار المزيفة وآثارها على السلوك والتصورات حول COVID-19.
يرجى الملاحظة أن المشاركة تطوعية وأن قرارك بالمشاركة أم لا لن يؤثر عليك بأي شكل من الأشكال. بالإضافة إلى ذلك ، سيتم التعامل مع جميع الإجابات التي تقدمها بشكل سري. سيتم استخدام نتائج هذه الدراسة لإبلاغ الاستجابة الوطنية للسيطرة على وباء المعلومات المتعلقة ب COVID-19.

لا توجد مخاطر مباشرة أو غير مباشرة مرتبطة بإكمال هذا الاستبيان ، وستبقى هويتك مجهولة.

بالإجابة على الاسئلة أنت توافق على المشاركة في الدراسة.

إذا كانت لديك مخاوف بشأن الدراسة أو أسئلة حول حقوقك كمشارك ، يمكنك الاتصال بمرصد السياسات العامة ومكتب الصحة عبر الهاتف على
+٩٦١ ٧١ ٧٢٩ ٧٩٥

* Required

1. ١- العمر *

Mark only one oval.

- ( ) ١٨-٢٤ سنة
- ( ) ٢٥ – ٤٠ سنة
- ( ) ٤١ – ٦٠ سنة
- ( ) ٦٠ سنة <

2. ٢- الجنس *

Mark only one oval.

- ( ) ذكر
- ( ) انثى
- ( ) أفضل عدم الاجابة

3. ٣- الحالة الاجتماعية *

Mark only one oval.

- [ ] اعزب/ عزباء
- [ ] متزوج/ متزوجة
- [ ] مطلق/مطلقة
- [ ] أرملة

4. ٤- أعلى شهادة مكتسبة *

Mark only one oval.

- [ ] ابتدائي
- [ ] ثانوية
- [ ] جامعة (بكالوريوس)
- [ ] جامعة (ماجستير)
- [ ] جامعة (دكتوراه)

5. ٥- الحالة التوظيفية *

Mark only one oval.

- [ ] موظف(ة)
- [ ] غير موظف(ة)
- [ ] متقاعد(ة)
- [ ] عاجز/ لست قادر على العمل

6. ٦- الجنسية

Mark only one oval.

- ( ) لبناني(ة)
- ( ) غير لبناني(ة)

7. ٧- ما هو المصدر الرئسي لمعلوماتك حول الفيروس؟ ضع علامة على كل ما ينطبق. *

Check all that apply.

- [ ] موقع مؤسسة الصحة العالمية (WHO)
- [ ] موقع وزارة الصحة اللبنانية
- [ ] الواتساب
- [ ] الفيس بوك و الانستغرام
- [ ] الانستغرام
- [ ] التويتر
- [ ] أخبار التلفاز
- [ ] أخبار الراديو

Other: [ ] ______________________

8. ٨- إلى أي حد تثق بأخبار فيروس الكورونا من المصادر المذكورة أدناه؟ *

Check all that apply.

	أبدا	نادرا	بعض الاحيان	غالبا
موقع مؤسسة الصحة العالمية (WHO)	[ ]	[ ]	[ ]	[ ]
موقع وزارة الصحة اللبنانية	[ ]	[ ]	[ ]	[ ]
الواتساب	[ ]	[ ]	[ ]	[ ]
الفيس بوك و الانستغرام	[ ]	[ ]	[ ]	[ ]
التويتر	[ ]	[ ]	[ ]	[ ]
أخبار التلفاز و الراديو	[ ]	[ ]	[ ]	[ ]

9. ٩- هل تعتقد أنك تعرضت لمعلومات خاطئة / أخبار كاذبة أو أو غير مؤكدة حول فيروس كورونا؟ *

Mark only one oval.

- أبدا *Skip to question 12*
- نادرا
- بعض الاحيان
- غالبا

Untitled Section

10. ١٠- في حال أجبت ب"نادرا"، "بعض الاحيان"، أو "غالبا" على السؤال ٩#، ما كان مصدر / مصادر الأخبار الكاذبة؟ *

Check all that apply.

- موقع مؤسسة الصحة العالمية (WHO)
- موقع وزارة الصحة اللبنانية
- الواتساب
- الفيس بوك و الانستغرام
- التويتر
- اخبار التلفاز و الراديو
- Other: ____

11. ١١- في حال أجبت ب"نادرا"، "بعض الاحيان"، أو "غالبا" على السؤال ٩#، ما هو نوع الخبر الكاذب؟ *

اذا اجبت "غير"، يرجى ان يحدد

Check all that apply.

- [ ] معلومات خاطئة حول طريقة انتقال الفيروس
- [ ] المبالغة في الخطر / الأضرار التي يسببها الفيروس
- [ ] نظريات المؤامرة حول كون الفيروس صنع اصطناعي / صنع انسان
- [ ] الأضرار الناجمة عن الكمامات أو الكلور / المعقمات
- [ ] انتشار الفيروس بتكنولوجيا الاتصالات المحمولة من الجيل الخامس (5G)
- [ ] فعالية بعض الأدوية و العلاجات
- [ ] العلاجات المنزلية من خلال التغذية
- [ ] غير متوفر

Other: [ ] ____________________

Untitled Section

12. ١٢- هل تشارك أو تنشر أو تضغط "اعجبتني" على الأخبار حول الفيروس على وسائل التواصل الاجتماعي؟ *

Check all that apply.

	نعم	لا
مشاركة	[ ]	[ ]
نشر	[ ]	[ ]
ضغط "اعجبتني"	[ ]	[ ]

13. ١٣- في حال أجبت ب"نعم"، إن المعلومات التي تم مشاركتها، نشرها أو الإعجاب بها تتعلق بـ: ضع علامة على كل ما ينطبق *

Check all that apply.

- [ ] الإجراءات الوقائية الشخصية (كارتداء الأقنعة تعقيم الأغراض ...)
- [ ] آخر المستجدات الحكومية حول الفيروس (كالتعبئة العامة والحالات الجديدة اليومية)
- [ ] آخر المستجدات العلمية حول الفيروس (كالضراوة والعدوى والمضاعفات الجسدية)
- [ ] نظريات المؤامرة حول الفيروس المتعلقة بأسباب سياسية
- [ ] لا أذكر المعلومات التي شاركتها او نشرتها
- [ ] غير متوفر

14. ١٤- هل تشارك أو تنشر المعلومات حول فيروس الكورونا دون التحقق من موثوقية المعلومات أو التحقق من ذلك مع مصادر الخبراء؟ *

Mark only one oval.

- ( ) أبدا
- ( ) نادرا
- ( ) بعض الاحيان
- ( ) غالبا

15. (١٥- أعتقد ان فيروس كورونا المستجد (اضغط على كل ما ينطبق *

Check all that apply.

	اوافق بشدة	اوافق	محايد\غير متأكد	لا اوافق	لا اوافق بشدة
يشكل خطر لك و لعائلتك	☐	☐	☐	☐	☐
مبالغ فيه في وسائل الاعلام (الخطورة, الوفيات و ما الى ذلك)	☐	☐	☐	☐	☐
مُركّب اصطناعيا في مختبر	☐	☐	☐	☐	☐
أداة تجارية لبيع اللقاحات و الادوية	☐	☐	☐	☐	☐
أداة للتحكم بالتركيب الديمغرافي للسكان (عبر تقليل عدد المسنين)	☐	☐	☐	☐	☐

لقاح ضد فيروس كورونا

16. ١٩- اذا كان هنالك لقاح ضد فيروس كورونا، هل تعتقد انك ستأخذ اللقاح؟ *

Mark only one oval.

- ⬭ نعم
- ⬭ لا
- ⬭ لست متأكد(ة)

17. شكرا على المشاركة

This content is neither created nor endorsed by Google.

Google Forms
